# Experimental and computational evidence that Calpain-10 binds to the carboxy terminus of Na_V_1.2 and Na_V_1.6

**DOI:** 10.1038/s41598-024-57117-8

**Published:** 2024-03-21

**Authors:** Luis Manuel Arratia, Juan David Bermudes-Contreras, Jorge Armando Juarez-Monroy, Erik Alan Romero-Macías, Julio Cesar Luna-Rojas, Marisol López-Hidalgo, Ana Victoria Vega, Absalom Zamorano-Carrillo

**Affiliations:** 1 Carrera de Médico Cirujano, FES Iztacala, UNAM, Av. de los Barrios 1, Los Reyes Iztacala, Tlalnepantla, Edo. Mex, Mexico; 2 Laboratorio de Biofísica Computacional, Doctorado en Biotecnología, SEPI-ENMH Instituto Politécnico Nacional, Av. Guillermo Massieu Helguera 239, Fracc. La Escalera, Ticomán, Gustavo A. Madero, 07320 Mexico City, Mexico; 3 Doctorado en Ciencias Biomédicas, FES Iztacala, UNAM, Av. de los Barrios 1, Los Reyes Iztacala, Tlalnepantla Edo, Mexico City, Mexico; 4 Maestría en Neurobiología, FES Iztacala, UNAM, Av. de los Barrios 1, Los Reyes Iztacala, Tlalnepantla Edo, Mexico City, Mexico

**Keywords:** Molecular neuroscience, Sodium channels, Computational biophysics, Membrane proteins

## Abstract

Voltage-gated sodium channels (Na_V_) are pivotal proteins responsible for initiating and transmitting action potentials. Emerging evidence suggests that proteolytic cleavage of sodium channels by calpains is pivotal in diverse physiological scenarios, including ischemia, brain injury, and neuropathic pain associated with diabetes. Despite this significance, the precise mechanism by which calpains recognize sodium channels, especially given the multiple calpain isoforms expressed in neurons, remains elusive. In this work, we show the interaction of Calpain-10 with Na_V_'s C-terminus through a yeast 2-hybrid assay screening of a mouse brain cDNA library and in vitro by GST-pulldown. Later, we also obtained a structural and dynamic hypothesis of this interaction by modeling, docking, and molecular dynamics simulation. These results indicate that Calpain-10 interacts differentially with the C-terminus of Na_V_1.2 and Na_V_1.6. Calpain-10 interacts with Na_V_1.2 through domains III and T in a stable manner. In contrast, its interaction with Na_V_1.6 involves domains II and III, which could promote proteolysis through the Cys-catalytic site and C2 motifs.

## Introduction

Voltage-gated sodium channels (Na_V_'s) are vital in initiating action potentials within excitable cells. Composing a central alpha subunit responsible for pore formation, these channels can also associate with one or two ß-subunits (ß1–4), which concurrently function as cell adhesion proteins^1^. The alpha subunits consist of approximately 2000 amino acids organized into four domains (I-IV) interconnected by intracellular loops. Both the N- and C-termini are situated intracellularly. Each domain encompasses six transmembrane segments (SI–S6) alongside a pivotal loop between S5 and S6 that forms the pore and constructs the selectivity filter. Within each domain, the S4 segments contribute to the voltage sensor and activation gate. The linkage between domains III and IV (linker III–IV) actively participates in the function of the inactivation gate. The linkers I–II, II–III, and the C- and N-termini seem more important for protein interactions. Nine isoforms of alpha subunits of sodium channels named Na_V_1.1 to Na_V_1.9 have been identified^2^. Since the discovery of the association of alpha subunits with auxiliary ß- subunits, our knowledge of the interactions of sodium channels with other proteins has grown to the point of considering that sodium channels are part of signaling macromolecular complexes^3^; however, the impact of these interactions on the channel function, or the function of its interacting partners is only starting to unveil. Such interactions may help to understand spatial and temporal fine-tuning of sodium channel expression. For example, Na_V_1.2 and Na_V_1.6 alpha subunits contain 86% amino acid similarity in the transmembrane domains^4^ and rather subtle differences in electrophysiological properties^5^. Also, they have a different subcellular distribution in neurons^6,7^, and controlling its expression during development and survival after birth seems essential. For example, in SNC8A null mice (medtg/medtg), the absence of Na_V_1.6 channels results in ataxia and early death of mice^8^, even though Na_V_1.1 and Na_V_1.2 fill the spots usually occupied for Na_V_1.6 and compensate for the lack of Na_V_1.6^9,10^.

Here, we show evidence that Calpain-10 binds the C-terminals of Na_V_1.2 and Na_V_1.6 in vitro and in silico. The Calpain family is a set of 14 non-lysosomal calcium-dependent proteases that perform limited proteolysis to modulate the function of its targets^11^. Calpains exhibit four domains: domain I in the N-terminus can be proteolyzed when the calpains are active, domain II possesses the Cys-catalytic site, and domain III exhibits C2 motifs. The C-terminus containing domain IV is very divergent. Depending on the version of this domain, calpains are classified as typical or atypical. Typical calpains exhibit a penta-EF-hand domain, while atypical calpains have different versions of the C-terminal domain^12^. Although typical calpains, such as Calpain-1 and -2, have been reported before to proteolyse sodium channels in isolated membranes^13^, it is unknown if other calpains can target sodium channels. Calpain-10 is considered an atypical calpain; its C-terminus was first known as domain T because of its homology to the nematode calpain TRA-3^14^. However, because both domain III and domain T present a beta-sandwich structure related to the calcium-binding C2 domain of phospholipase C, domain T has received several names: domain III’, C2L or C2-like, and CBSW domain (calpain-beta sandwich). In this work, after demonstrating the physical interaction between Calpain-10 with Na_V_1.2 and Na_V_1.6, we offer a structural and dynamical hypothesis to explain the interactions in atomistical terms. The docking analysis and molecular dynamics simulation showed that the C-terminus might be relevant for Calpain-10 to dock at the C-terminus of sodium channels, suggesting this process as a regulatory mechanism of neural activity.

## Results

### Experimental evidence of binding between Calpain-10 and Na_V_’s C-terminals

**Figure 1:**
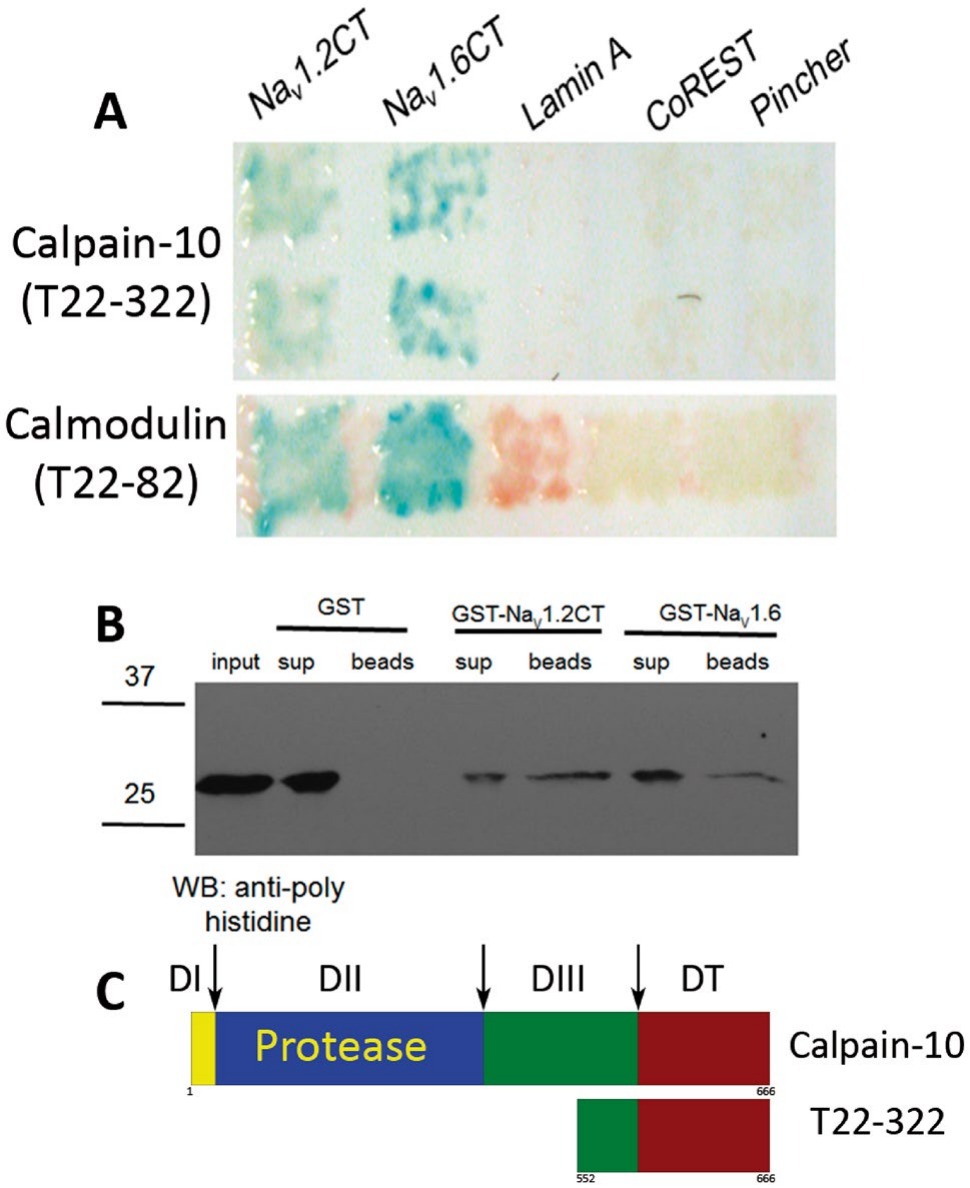
Calpain-10 binds to the C-termini of sodium channels Na_V_1.2 and Na_V_1.6 through domains III and T in Y2H assay. (**A**) L40 yeast expressing Calpain-10 were mated with AMR70 cells previously transfected with plasmids encoding Na_V_1.2 CT, Na_V_1.6 or a series control proteins (Lamin A, CoREST and Pincher). The interaction between transfected proteins induced expression of ß-galactosidase reporter gene. The presentation of ß-galactosidase produces blue color in presence of the hydrolyzable substrate X-gal. Only those cells expressing Calpain-10 and Na_V_1.2CT or Na_V_1.6CT could hydrolyze X-gal. As a positive control, we show the same experiment with cells expressing calmodulin. (**B**) In vitro binding of His_6_Calpain-10 to the C-terminus of Na_V_1.2 and Na_V_1.6. Recombinant proteins GST, GST-Na_V_1.2CT or Na_V_1.6CT, bound to glutathione-beads, were incubated with recombinant His_6_-Calp10. After extensive washing, the proteins bound to glutathione-beads were analyzed by western blot with an anti-poly-Histidinde antibody. A single band of ~ 29 kDa, matching the expected molecular weight of the His6-Calpain10, was identified, indicating that it is able to bind to GST-Na_V_1.2CT and GST-Na_V_1.6CT but does not bind to GST alone. Full length blots are shown in Supplementary Fig. S3. (**C**) Scheme shows the fragment of Calpain-10 identified initially as a protein capable of binding dependent sodium channels voltage corresponding mainly to the domain T. Numbers under scheme 2D of Na_V_'s corresponding at alpha helix number.

Interactions of sodium channels with proteins have proven capable of regulating channel function. We have reported before that over 50 proteins are able to bind the C-terminus of Na_V_1.2 in the yeast-two hybrid assay^15^. We screened a cDNA library from mouse brain, from which we recovered binding partners previously reported, like FHF4, Calmodulin, and mSin3B. Here we report another binding partner found in this screening. We found two clones containing 215 aa of Calpain-10 (amino acids 452 to 666), including the last 36 aa of domain III and the whole of domain T. In Fig. 1A, we show that cells expressing Na_V_1.2 or Na_V_1.6, but not negative controls (LaminA, CoREST, and Pincher), exhibit ß-galactosidase activity as a result of the interaction between the C-terminus of these channels and domains III and T of Calpain 10 encoded in clone T22–322. Another clone (T22–82) from the same YTH screening expressing calmodulin, a well-known interacting protein of sodium channels, is also shown as a positive control. To further verify this interaction, the plasmid recovered from yeast was subcloned to produce a His6-tagged version of Calpain 10 with a calculated molecular weight of 28.9 kDa (His6-Calpain10) to be used in a GST-pull down assay. Once again, GST-tagged versions of Na_V_1.2 and Na_V_1.6 could retain the His6-Calpain10, while GST alone did not (Fig. 1B). Calpain 10 scheme shown in Fig. IC depicts clone T22-322, including part of DIII and DT.

### Modeling and docking analysis

Explaining molecular mechanisms based on a peptide interaction model is valuable as it can offer an atomistic description of cellular processes, such as the role of peptides as toxins^16^, conjugated peptides with anticancer activity^17^, or antimicrobial peptides^18^, to name a few. In our group, we have jointly used homology modeling, docking, and molecular dynamics to model the interaction of an immunogenic peptide with the Major Histocompatibility Complex II (MHC II)^19^ or even RNA–Protein interaction^20^.

Here, we first generated 3D models for the C-terminus of Na_V_1.2 and Na_V_.16 using automatically selected templates by the comparative modeling method implemented in the Robetta server^35^. Then, the models were analyzed using PROCHECK, which revealed that 88.9% of residues in Na_V_1.2 and 90.2% of Na_V_1.6 were in the most favored regions of the Ramachandran plot, and 10.1% and 8.8%, respectively, of residues were in the additional allowed regions. It is worth mentioning that the most distal part of both sequences are de novo predictions since the available templates cover roughly the first half or less of the sequence. It is also important to remember that the C-terminus of sodium channels becomes more variable among isoforms towards the second half of the sequence (see Supplementary Fig. S4).

Another important novelty of this work is the use of the whole Calpain-10 model to study protein–protein interaction since previous studies on Calpain-10 molecular dynamics and docking have focused on the catalytic domain and its interaction with inhibitors, such as calpastatin^21,22^. Instead, the roles of domains III and T have been overlooked despite their potential for binding targets. Here we used a template from Alphafold and refined it with Robbeta. The resulting model has a 91.7% of amino acids within the most favored region and 7.9% in the allowed region.

**Figure 2:**
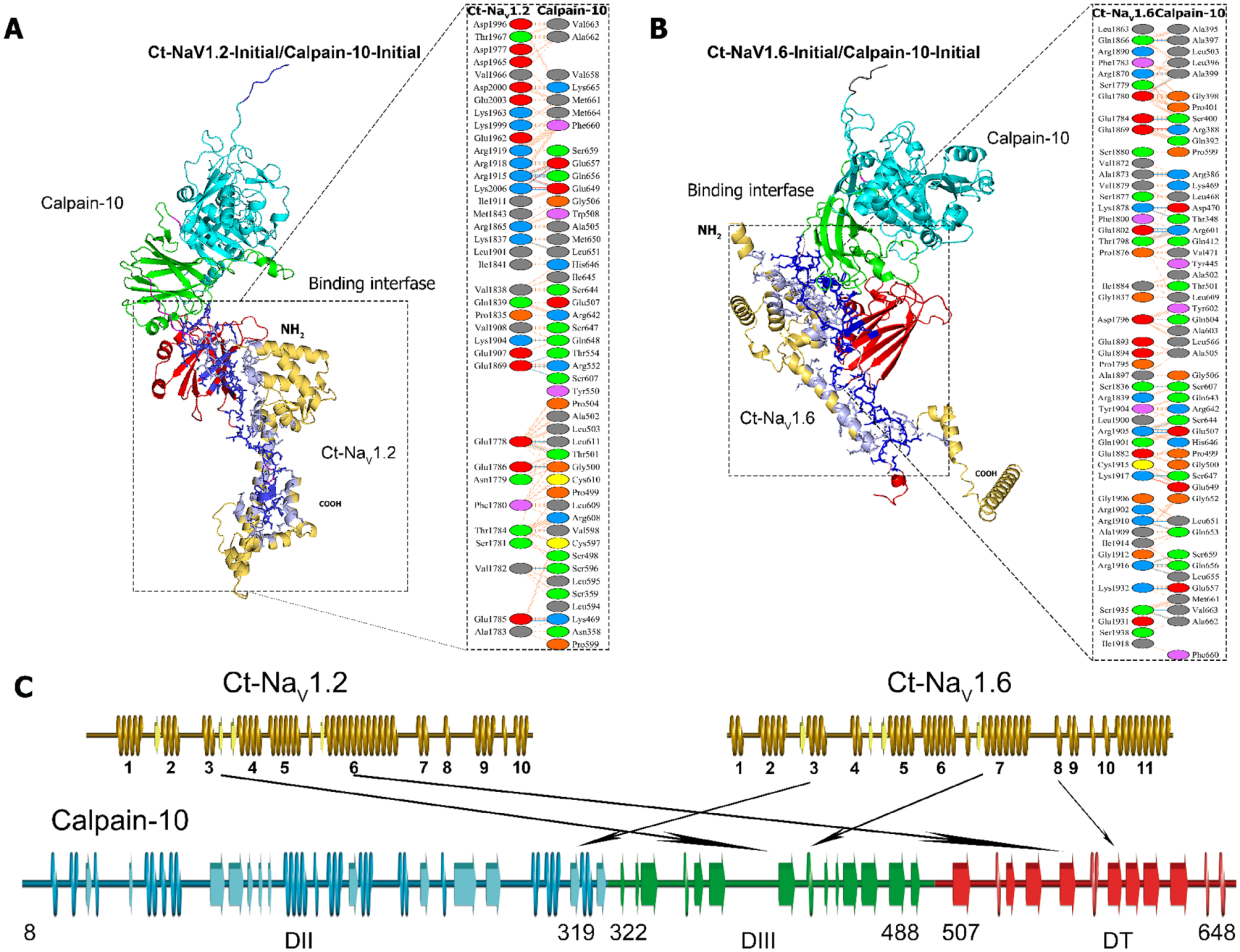
Molecular recognition of C-terminus of Na_V_1.2 and Na_V_1.6 with Calpain-10. Docking of Calpain-10 to Na_V_1.2CT (**A**) and Na_V_1.6CT (**B**) is shown as a 3D model. The domains of Calpain-10 were in different colors: Domain II in Cyan color, domain III in green color, and domain T in red color. Na_V_1.2 and Na_V_1.6 structure is shown in yellow (non interacting surfaces) and dark blue, while in blue color are the interactions. Amino acids relevant to docking on both proteins are listed right into the 3D models. Hydrogen bonds (blue solid lines), salt bridges (red solid lines), and non-bonded contacts (orange dashed lines) are shown. Amino acids color code: positive (blue), negative (red), neutral (green), aliphatic (gray), aromatic (purple), Pro and Gly (orange), and Cys (yellow). Amino acids of Na_V_1.2 and Na_V_1.6 correspond to amino acids 1778-2006 of Na_V_1.2 (Uniprot: B1AWN6; NCBI ID: BAC27748.1) and 1765-1978 of Na_V_1.6 (Uniprot: Q9WTU3; NCBI ID: NP_035453.2). (**C**) Secondary structure schematic highlights the regions more relevant to the interaction between the C-terminal ends of the Na_V_1.2 and Na_V_1.6 channels and the characteristic domains of Calpain-10.

The HDock and Cluspro 2.0 programs provided the complexes of Calpain-10 with the Na_V_1.2CT and Na_V_1.6CT, using a cutoff of 5 Å. Later, the interactions in each complex were found by PDBsum. We analyzed the 20 more energetically favorable models, ten from each docking program. Then, we determined the most frequent interacting amino acids to obtain a more representative interaction model. Noteworthy, all of the docking poses of Na_V_1.6 were different from those of Na_V_1.2. This result was unexpected, given the similarity of a good part of the amino acid sequences (see Supplementary Fig. S4). Figure 2A,B presents a representative model that summarizes these interactions.

### Docking of Na_V_1.2CT with Calpain-10

We docked the C-terminus of Na_V_1.2 with amino acids 452 to 666 of mouse Calpain-10 and analyzed the amino acids that participate in the interaction between these two proteins. The PDBsum interaction analysis revealed that eight salt bridges, 23 hydrogen bonds, and 318 nonbonded contacts were formed between Na_V_1.2CT with Calpain-10. In these interactions, 36 residues of Na_V_1.2CT and 47 of Calpain-10 are involved, for which four double hydrogen bonds were formed between Thr1784–Val598, Glu1785–Lys469, Arg1915-Glu657 and Asp2000-Lys665. The eight salt bridges are formed between residues Glu1785-Lys469, Glu1869-Arg552, Arg1915-Glu649, Arg1915-Glu657, Arg1918-Glu657, Asp2000-Lys665, Lys2006-Glu649, and Lys2006-Glu657 (Fig. 2A). Of the 318 nonbonded contacts, 128 occurred on the atoms of the first nine amino acids of the C-terminus of Na_V_1.2; at these same residues, two salt bridges occurred. The eight salt bridges form hydrogen bonds and nonbonded contacts, suggesting an important role in promoting this interaction. The docking score for the Na_V_1.2CT/Calpain-10 complex was − 227.56 and a ΔG of -20 kcal/mol with a confidence score of 0.8251 (Table 1).

**Table 1:** Molecular docking scores of Na_V_1.2CT and Na_V_1.6CT with Calpain-10.

Protein–protein complex	Docking score	Confidence score	ΔG (kcal mol^−1^)
Na_V_1.2CT/Calpain-10	− 227.56	0.8251	− 20.0
Na_V_1.6CT/Calpain-10	− 308.57	0.9597	− 21.1

Protein–protein interactions, the number of interfacial contacts, and non-interacting surfaces were used to calculate the table binding affinity of proteins as Vangone & Bonvin, 2015.

### Docking of Na_V_1.6CT with Calpain-10

Following the same strategy depicted above, we analyzed the interaction between Calpain-10 and Na_V_1.6. The interaction analysis revealed that the Na_V_1.6CT/Calpain-10 interaction is constituted of 11 salt bridges, 36 hydrogen bonds, and 407 nonbonded contacts, in turn, formed by 46 residues from the C-terminus of Na_V_1.6 and 57 residues of Calpain-10 (Fig. 2B). The hydrogen bonds formed by the Na_V_1.6CT/Calpain-10 complex include five double bonds between the residues Glu1784-Ser400, Arg1905-Glu507, Glu1802-Arg601, Arg1910-Leu651 and Arg1916-Gln-656. On the other hand, 9 of the 11 salt bridges also form hydrogen bonds and non-bonded contacts. These are comprised between Glu1869-Arg386, Thr1798-Arg601, Ala1873-Arg386, Glu1869-Arg388, Arg1905-Glu507, Glu1802-Arg601, Arg1907-Glu507, Lys1917-Glu649 and Lys1932-Glu657, while Glu1869-Arg388 and Lys1917-Glu649 form only salt bridges and non-bonded contacts. The docking score for the Na_V_1.6CT/Calpain-10 complex was − 308.57 and a ΔG of − 21.1 kcal/mol with a confidence score of 0.9597 (Table 1).

Notably, a key distinction arises in the interaction mechanisms of Na_V_1.2CT/Calpain-10 and Na_V_1.6CT/Calpain-10. While the former utilizes its initial nine N-terminal residues for interaction, the latter does not involve these residues. Furthermore, although in both cases the interaction domain encompasses the alpha helix 6, Na_V_1.6CT/Calpain-10 also involves five extra amino acids (GFICRK) unique to the Na_V_1.6 sequence, further demarcating the divergent characteristics between these channels. A schematic representation of these differences is shown in Fig. 2C.

### Geometrical and dynamical parameters of Calpain-10, Na_V_1.2CT/Calpain-10, and Na_V_1.6CT/Calpain-10

**Figure 3:**
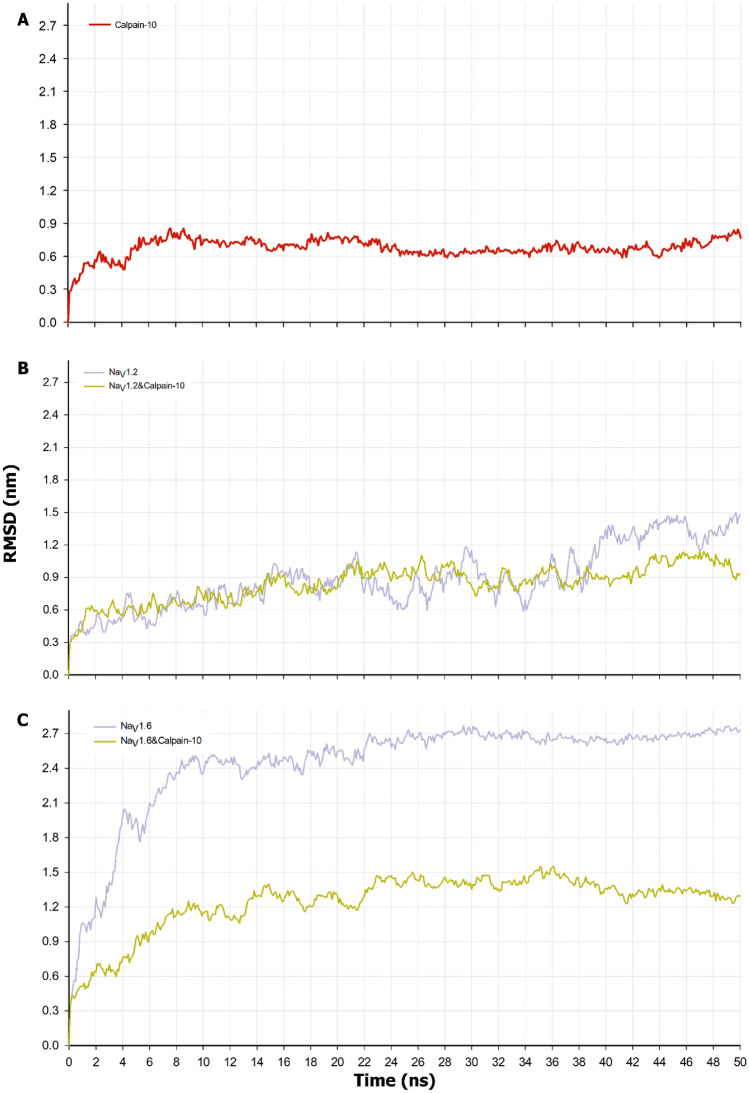
RMSD of Calpain-10 and bound to Na_V_1.2CT or Na_v_1.6CT. (**A**) Calpain-10 RMSD value remains stable through the simulation. (**B**) Na_V_1.2CT RMSD values are more stable when in complex with Calpain-10 than for Na_V_1.2 alone; (**C**) RMSD values for Na_V_1.6CT are higher than those observed for Na_V_1.2 suggesting a more flexible structure. Again a reduction of the RMSD for the Na_V_1.6/Calpain 10 is noticeable, indicating a more stable structure.

To investigate the protein stability, residue fluctuations, and dynamic behavior, 50 ns of MD simulations of the Calpain-10, Na_V_1.2CT/Calpain-10, and Na_V_1.6CT/Calpain-10 were performed (Fig. 3). The RMSD of the backbones, as a parameter of structural stability during the simulations, was calculated and compared with the reference structure at t = 0. As shown in Fig. 3, Calpain-10 alone seems stable (RMSD = 0.677 ± 0.087 nm), and Na_V_’s gain stability through its interaction with Calpain-10. RMSDs for Na_V_1.2CT/Calpain-10 complex and Na_V_1.2CT are shown in Fig. 3B. After an initial increase of RMSD during the first 12 ns, both simulations reach a fluctuant stability around 0.842 ± 0.168 nm and 0.892 ± 0.286 nm, respectively. This result indicates minor conformational changes in Na_V_1.2CT when bound to Calpain-10. It also indicates a stable binding over 40 ns of Na_V_1.2/Calpain-10; actually, the complex seems more stable than the Na_V_1.2CT alone beyond this point. Na_V_1.6 presented higher RMSD values (2.44 ± 0.445 nm) than Na_V_1.2 and also higher values than Na_V_1.6CT/Calpain-10 complex (1.242 ± 0.248 nm) and Calpain-10 alone (Fig. 3B). However, its binding with Calpain-10 resulted in lesser RMSD values for the complex Na_V_1.6CT/Calpain-10 compared to Na_V_1.6CT alone, roughly by 50%. This result indicates that the interaction of the C-terminus with Calpain-10 provides them with greater stability. Furthermore, the Na_V_1.2CT/Calpain-10 complex starts from a more stable conformation than the Na_V_1.6CT/Calpain-10 complex.

The differences in RMSD behavior are consistent with the evolution in the number of hydrogen bonds and the number of electrostatic interactions at the initial structure and the final of the NaVs-Calpain-10 complexes. For NaV1.2/Calpain-10, although there were fluctuations in quantity, the number of these remained relatively constant when the initial and final structures were compared, approximately 620 HBonds and 8 to 6 salt bridges. However, for NaV1.6/Calpain-10, the HBonds were initially 650 and were reduced to 620 in the final structure, and the number of salt bridges almost doubled from 7 to 13 during the simulation.

**Figure 4:**
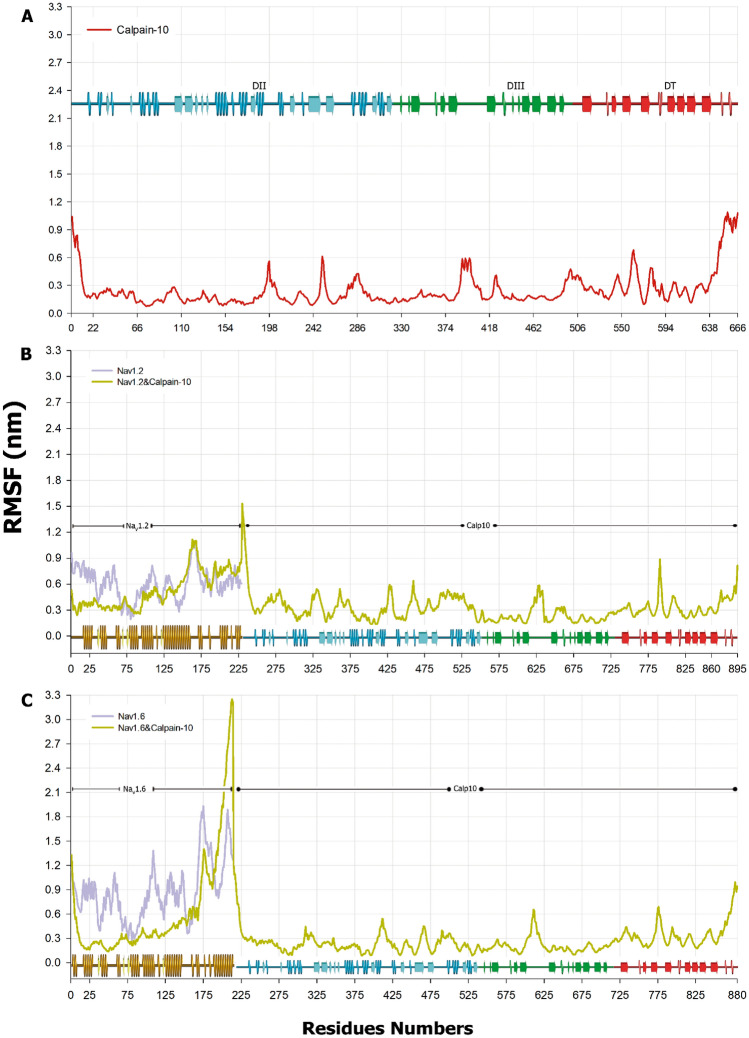
RMSF of Calpain-10 and bound to Na_V_1.2CT or Na_V_1.6CT. Calpain-10 RMSF is presented in (**A**). Notice a more pronounced variation towards the N- and C-termini. Consistent with the RMSD, Na_V_1.2CT(**B**) individual residue fluctuations are smaller compared with that of Na_V_16CT(**C**). In both cases these fluctuations are reduced by interaction with Calpain-10 (olive green). Notice that Calpain-10 RMSF profile remains basically unchanged by the interaction with either Na_V_1.2CT or Na_V_1.6CT.

The RMSF of the Cα atoms was calculated to analyze the residues' fluctuations. To clarify how the results are shown, we must mention that Fig. 4B,C show a number greater than 800, representing the sum of residues in each C-terminus of Na_V_’s with Calpain-10. For Calpain-10, as observed in the RMSF, its structure remains stable throughout the MD; we found three peaks that exceed 0.4 nm. The first is in domain III (Val379-Asp410), while the second and the third are in the T domain between Ile551-Leu595 (Fig. 4A). In Fig. 4B, the RMSF of the complex Na_V_1.2/Calpain-10 is shown. The RMSF values for Na_V_1.2CT indicate more flexibility than the observed for Calpain-10 since most structures show values above 0.4 nm. However, these values decrease when it is in complex with Calpain-10, except for the α6 helix and the zone of most significant divergence between sodium channels (Ser153-Leu179), which can be considered a zone of important movements for the binding with Calpain-10 (Fig. 4c).

The RMSF for Na_V_1.6CT shows a profile similar to that observed for Na_V_1.2CT up to Leu145, consistent with the 93% similarity in amino acid sequence between them; however, the sequences are divergent from this residue. The residues with the highest flexibility in the Na_V_1.6 complex are located from Ile150 and up to Cys214. Interestingly, this region is involved in the interaction with Calpain-10 (Fig. 4C) and exhibits residues forming hydrogenous bridges, unconnected contacts, and salt bridges (Lys168-Glu657; Arg152-Glu657; Lys168-Lys665) (Fig. 2B). Thus, the interaction with Calpain-10 stabilizes the sodium channel structure mainly within the highly conserved region, but it does not reduce the peaks observed in the divergent region. It seems to increase the mobility of the last α-helix in Na_V_1.6CT (Pro188-Cys214). Sodium channels are reported to bind calmodulin through an IQ motif in the α6 helix of Na_V_1.2 C-terminus. Although the binding surface for Calpain-10 does not overlap with the IQ motif, the surface involves part of the alpha helix needed to bind calmodulin. Therefore, some competition might be expected. In summary, at the far left of the graph of 4B and C, we observed a comparison of the Na_V_1.2CT and Na_V_1.6CT free or bound to Calpain-10. Thus, we conclude that this interaction stabilizes the C-terminal ends, significantly affecting Na_V_1.6CT.

### Secondary structure evolution

The evolution of the secondary structure as a function of time and the number of residues for Calpain-10, Na_V_1.2CT, and Na_V_1.2CT/Calpain-10 are shown in Supplementary Fig. S1. The secondary structure of Calpain-10 is well preserved during the MD simulation (Supplementary Fig. S1A) except for the amino and carboxyl-terminal ends. Na_V_1.2CT loses part of the α6 helix, which contains the IQ domain important for calmodulin binding (Supplementary Fig. S1B). Conversely, in the Na_V_1.2CT/Calpain-10 complex, the N- and C-terminus of α6-helix seem to be structurally more robust; the same is more evident in the α-helices 4, 5, suggesting more structural stability (Supplementary Fig. S1C). Also, complex Na_V_1.2CT /Calpain-10 induces partial unfolding of Calpain-10 in its α-helices 4,5, 7 (DII), and in the β-sheets 18 and 19 (DIII), while aid to stabilize both the N-and C-termini. In contrast to Na_V_1.2CT, the MD of Na_V_1.6CT alone (Supplementary Fig. S2B) shows that its α-helices are more preserved along the simulation. However, the interaction with the CT’s seems to destabilize the structure of Calpain-10, specifically α-helices 9, 16 y 17 (Supplementary Fig. S2). In contrast, the N- and C-terminus of Calpain-10 tend to be more stable when in the complex with Na_V_1.6, although domain II α-helices and ß-strands remain the same. The loop joining PC1 and PC2 (catalytic subdomains) seem to be less rigid.

These results indicate that the secondary structure of Calpain-10 undergoes more changes when it is in complex with Na_V_1.2 than with Na_V_1.6, suggesting a different behavior with its targets. Based on the results of the RMSD, RMSF, and secondary structure evolution and describing the effect on Calpain-10 due to its binding to Na_V_CTs, we can observe that the interaction with Na_V_1.6 does not alter its secondary structure, even though Calpain-10 has a significant stabilizing effect on this C-terminus. Interestingly, consolidating Calpain-10's interaction with Na_V_1.2CT costs the protein a portion of its secondary structure.

## Discussion

Here, we show evidence that Calpain-10 binds to the carboxyl termini of Na_V_1.2 and Na_V_1.6. First, the yeast two-hybrid assay found that a region comprising 36 amino acids of domain III and the whole of domain T of Calpain-10 could interact with the C-termini of sodium channels Na_V_1.2 and Na_V_1.6. The ability of this region to bind sodium channels was then confirmed in a pull-down assay. Given these experimental findings, we use 3D homology models to predict macromolecular docking and molecular dynamics of the interaction. Thus, we found that a surface spanning the sixth alpha helix of Na_V_1.2 and the seventh helix in Na_V_1.6 may provide several interactions with domains III and T of Calpain-10.

A novelty of this work is the use of the whole Calpain-10 model to study protein–protein interaction since previous studies on Calpain-10 molecular dynamics and docking have focused on the catalytic domain and its interaction with inhibitors, such as calpastatin^21,22^. Instead, the roles of domains III and T have been overlooked despite their potential for binding targets. We found that Calpain-10 binding of the carboxy terminus of Na_V_1.2 and Na_V_1.6 has a favorable free energy consistent with the results of the GST-pulldown assay. Likewise, the RMSD analysis indicated that the carboxy-terminus of both Na_V_1.2 and Na_V_1.6 become more stable upon interaction with Calpain-10, which explains why the complex lasts long enough to recruit the transcription machinery to the reporter genes HIS3 and beta-galactosidase in the YTH assay.

Recent crystallographic studies of heterologously expressed channels often resort to the removal of linkers I-II, II-III, and C-terminus to improve expression, thermostability, and sample homogeneity compared with full-length wild type channels^23,24^. Although it results in a channel with biophysical properties similar to full-length channels, the need to remove intracellular amino acid chains that account roughly 50% of the protein remarks the importance of these regions to downregulate expression of channels in non-excitable cells and the need to study their interactions with other proteins. Establishing the interactions of the C-terminus with multiple proteins may help refine the search for an allosteric modulator specific to the interacting partner, as already assayed for FGF14^25^. We selected Na_V_1.2 and Na_V_1.6 to search for new binding partners because these two channels are the most abundant voltage-gated channels in the adult brain, and despite their overall similarity, there is a clear segregation of their distribution at subcellular level in neurons^6,7^. We selected the C-terminus region as bait in the Y2H assay because of its divergence among sodium channels, hoping to find binding partners that showed a preference for a particular type of sodium channels. However, we have found that almost every binding partner for Na_V_1.2 can also bind Na_V_1.6. Nevertheless, our analysis indicates the interaction between Calpain-10 and Na_V_1.6 differs from that with Na_V_1.2, possibly due to differences in the c-terminal ends, despite the high identity between the Na_V_’s (Supplementary Fig. S4).

A most interesting observation is that two amino acids (Arg 1870 and Leu 1863) of Na_V_1.6 relevant to the interaction with calpain also have naturally occurring variants associated with epilepsy^26–28^. Mainly, Arg1872 of human Na_V_1.6, Arg1870 in mice (see Supplementary Fig. S5) has been reported to be a hotspot for missense mutations^26–28^ related to several forms of epilepsy, including Arg1872Trp, Arg1872Gln and a mutant that produces a truncated C-terminus. The impact of these mutations on binding not only Calpain-10 but other known interactors, such as calmodulin and FGF14 (Fibroblast Growth Factor 14), may help better understand the changes in excitability and its relationship to the survival of these cells. The binding of FGF14 to the C-terminus region of Na_V_1.6 has also been studied to an atomistic level^29^. Notably, charged residues such as Arginine and Glutamate, and an uncharged polar residue such as Serine participated significantly in the interaction, as reported by Ali et al.^29^, for the FGF14/Na_V_1 complex. 6. We found three residues relevant to Calpain-10 binding also interact with FGF14 (Ser1836, Arg1890 and Ile1884). Therefore, some degree of competition to bind this region may be expected, as it happens with calmodulin, which has also been found in association with the C-terminus of all types of sodium channels through an IQ motif that, in Na_V_1.6, becomes an LQ motif. In fact, in our YTH assay, calmodulin was the most frequently binding partner found (over 400 clones!), followed by mSin3B and FGF14 (See Supplementary Table S1).

Early experiments have shown that calpains do not affect the activity of the kinetics of sodium channel Na_V_1.5 as other proteases do^30^, while in chromaffin cells (expressing mainly Na_V_1.7), the calpain inhibitor calpastatin prevented a Ca-induced reduction of [3H]STX binding sites^31^. These early findings suggest that not all sodium channel alpha subunits interact with calpains. The docking analysis herein indicates that the first nine residues of Na_V_1.2 (ENFSVATEES) are relevant for the interaction with Calpain-10. Surprisingly, although this sequence is also present in Na_V_1.6, our modeling predicts that this set of residues is not involved in the interaction with Calpain-10, instead a cluster of residues from the most variable region 1912–1918 (GFICRK) becomes highlighted (see Supplementary Fig. S4), especially Arg1916 establishes four hydrogen bonds to residues 655, 656 and 659 in Calpain-10 domain T. This observation also highlights the relevance of the divergent sequences found in the carboxy-terminus of the sodium channel, where the sequence of amino acids is highly conserved through the first 180 amino acids, but the rest of the amino acid chain is highly divergent both in length and sequence among sodium channels. Further analysis of every Na_V_ would be necessary to determine if Calpain-10 can regulate the members of the sodium channel family differentially.

On the other hand, little is known about the physiological effects of partial proteolysis of sodium channels by calpains. In vitro studies on calpain-mediated proteolysis have led to conclude that calpains targets brain sodium channels, or more specifically Na_V_1.2 and Na_V_1.1, in models of injury, such as brain injury, stretch injury, and ischemia^13^. However, these models do not allow the detail of the particular contribution of each calpain expressed in the neurons, which would be essential to define since there are at least five calpains expressed in the mouse brain, calpains 1, 2, 5, 7, and 10^32^. Other efforts employ purified Calpain-1 to assess its ability to proteolyse sodium channels, but more information should be obtained regarding other calpains. Additionally, these studies use calcium concentrations that are hard to reach in physiological conditions^13,33,34^. In a model of spinal cord injury (SCI), an increase in sodium persistent current could be associated with partial cleavage of sodium channels by calpains and generation of a ~ 120 kDa fragment^34^. Similarly, Na_V_1.6 exposed to Calpain-1 degradation, in HEK293 cells, increased persistent current, indicating a gain of function after proteolysis. Also, some authors have suggested that the 120 and 160 kDa fragments of Na_V_1.2 produced by calpains remain associated for some time before being withdrawn from the membrane^13^, but its effect on current is unknown. However, as we now realize, Calpain-10 might interact differently with either Na_V_1.2 or Na_V_1.6 and thus differ from the interaction with Calpain-1. Therefore, more studies should be performed before drawing any conclusions.

The interaction between Calpain-10 and sodium channels may be especially interesting in the context of diabetes and the development of neuropathic pain, a common complication of diabetes. In most ß-cell types studied to date, Na_V_1.3 and Na_V_1.7 appear to play different roles, and in human ß-cells alpha-subunits Na_V_1.7 and Na_V_1.6 are present^35^. Also, it has been reported that sodium channels inhibitor carbamazepine positively affects *β*-cell survival and insulin production^36^. On the other hand, some gene variants of Calpain-10 have been implicated as risk factors for the development of type 2 diabetes^37^, and diabetic patients exhibit an increase in Calpain-10 expression in B-cells^38^. A down-regulation of sodium channels activity, mediated by Calpain-10 proteolysis, may be part of the standard mechanisms of regulation of ß-cells excitability, thus indirectly regulating calcium entry through voltage-gated calcium channels and insulin release. Interestingly, Calpain-10 expression is also elevated in glial cells in patients with Alzheimer's disease^39^, where sodium channel Na_V_1.6 is the predominant isoform^40^. Docking analysis indicates that the binding between Calpain-10 and Na_V_1.2 or Na_V_1.6 is different, making a contact surface rather unique that may be helpful later on to design inhibitors targeting specific protein–protein interactions. Docking analysis with other sodium channels may help to refine drug design. Likewise, docking analysis of other intracellular chains may further our understanding of the interaction between sodium channels and calpains.

## Methods

### Yeast two-hybrid assay (Y2H)

The procedure followed for Y2H screens was described before by Vega et al.^15^. For the initial screen, yeast cells of strain L40 were co-transfected with the bait plasmid pSN12CT together with a commercial plasmid library derived from adult mouse brain cDNA in fusion with GAL4 activation domain (Clontech, catalog #ML4008AH). Transfected cells were plated on -his -leu -trp medium, on which L40 yeast cells are able to grow only if they contain both bait and prey plasmids and if the LexA/GAL4 activator is formed by interaction of bait and prey proteins. After 3 days at 30 °C, white colonies > 2 mm in diameter were picked and replated on the triple-deficient medium. Colonies were tested for LexA-driven β-galactosidase activity, after 3 rounds of replating and testing for ß-galactosidase activity, positive colonies were released of trophic pressure to reproduce the bait plasmid, by plating them in -Leu medium, resulting prey-only cells. To determine specificity of prey interaction with sodium channels, these cells were then crossed with mating-proficient AMR70 yeast cells transfected with negative control bait-plasmids, or with the pSN12CT or pSN16CT plasmid to reconfirm the original interaction. Colonies were then tested for β-galactosidase activity, as an index of bait-prey interaction. Afterwards, prey plasmids were recovered and sequenced.

### Plasmids

Plasmids pSN12CT and pSN16CT^15^ contain bases 5512–6198 (NM_001099298.3) and 5293–5934 (AF049617) of Na_V_1.2 and Na_V_1.6 coding regions, corresponding to amino acids 1778-2006 and 1765-1978 of the respective α-subunits. They express the C-terminus of Na_V_1.2 or Na_V_1.6 in frame with the DNA-binding domain of LexA in two-hybrid bait plasmid pSTT91. Bait plasmids were confirmed not to have endogenous GAL4 activity when expressed in yeast. Negative-control bait plasmids encoding LexA-LaminA, LexA-CoREST and LexA-Pincher. C-terminal cDNAs were also subcloned into pGEX to produce GST-tagged versions of Na_V_1.2CT and Na_V_1.6CT (plasmids pGN12CT and pGN16CT). Two prey plasmids recovered from yeast matched bases 1819–2877 of mouse Calpain 10 (NM_011796.2), encoding amino acids 452–666 of sequence NP_035926.2. This partial calpain-10 sequence was subcloned into pET-28 plasmid to produce a 6His-tagged version (pEcalp10 plasmid). Full-length mouse Calpain-10 cDNA was amplified by PCR from a plasmid obtained from the Mammalian Gene collection, MGC-13731, IMAGE: 4159512, EcoRI and BamHI sites were introduced into the amplification primers to clone into pEGFP-C1(Clontech).

### Glutathione S-transferase (GST) pull-down assay

Detailed explanations about the GST-pull down assay have been published before^15^. GST, GST-Na_V_1.2CT, and GST-Na_V_1.6CT proteins were obtained in *E. coli* BL21 cells transformed with pGEX, pGN12CT, and pGN16CT, respectively. Cells were induced with 1 mM IPTG for 4 h at 37 °C. Then lysed by snap freezing and digestion with lysozyme, DNA was degraded by sonication. Glutathione sepharose beads were incubated with the lysate for 1 h at 4 °C and then washed extensively with ice-cold binding buffer (50 mM Tris–HCl, pH 7.5, 120 mM NaCl, 2 mM EGTA, 0.1% triton X-100, 2 mM DTT). BL21 cells transformed with pECalp10 were used to produce a His_6_-tagged recombinant Calp10 (His_6_-Calp10) expressing a, and then lysed as described above. Beads preloaded with GST, GST-Na_V_1.2CT, or GST-Na_V_1.6CT were incubated with the Calpain lysate for 1 h at 37 °C, in presence or absence of calcium. Beads were washed extensively with binding buffer and resuspended in 2X Laemmli reducing sample buffer (RSB). Pulled-down proteins were resolved by standard SDS-PAGE, electro blotted, and then subjected to western blot with mouse anti-polyhistidine antibody (1:6000, Sigma Aldrich H1029) and HRP-conjugated anti-mouse IgG secondary antibody (Santa Cruz Biotechnology). Blocking and incubation with antibodies were carried out in blotto (20 mM Tris–HCl, 150 mM NaCl, 4% non-fat milk, pH8.0). Immunoreactive bands were visualized by chemiluminescence and captured in film. After exposure, the film was hand-developed and digitized with a Canon PowerShot G5. Digitized images of three different experiments, including the blot shown in Fig. 1, are presented in full length in Supplementary Fig. S3.

### Molecular modeling

3D models of the mouse Calpain-10 (with NCBI Ref. Seq NP_035926.2), Na_V_1.2, and Na_V_1.6 (BAC27748.1, NP_035453.2 respectively) were constructed by homology in the Robetta server^41^. The mouse Calpain-10 model in full (666 amino acids), based on a mouse Calpain-10 structure (ID: AF-Q9ESK3-F1) predicted in Alphafold^42^, while Na_V_1.2 and Na_V_1.6 were modeled only by the cytosolic C-terminals, Na_V_1.2 model includes 229 amino acids (1778-2006), and Na_V_1.6 includes 214 amino acids (1765-1978), corresponding to the same amino acids expressed in the Y2H and the GST-pulldown assays. The structures of C-termini of Na_V_1.2 and 1.6 were constructed using partial crystallographic models of the human Na_V_1.2, 1.4, and 1.5 C-termini as templates (PDB: 4JPZ, 6MBA, and 2KBI), selected automatically by Robetta. To assess the geometry and restriction violations of these protein structures we use PROCHECK free software^43^, thus we calculate the degree of agreement of the model structures with the experimental data, and the quality of its geometric properties.

Protein–protein coupling of Na_V_1.2CT and Na_V_1.6CT with Calpain-10 were performed with ClusPro 2.0 and the HDOCK server (http://hdock.phys.hust.edu.cn/), which is based on a search ensemble through homology, template-based modeling, structure prediction, macromolecular docking, biological information incorporation, and data-management works for a robust and rapid protein–protein coupling. With input information on the receptor and ligand molecules (either amino acid sequences or structures from the protein databank), the server automatically predicts their interaction through a hybrid template-based and templateless-matching algorithm^44^. The HDOCK server globally samples all possible binding modes between the two proteins through an algorithm based on the fast Fourier transformation^45^. Then, all sampled binding modes were evaluated using the iterative knowledge-based scoring function ITScorePP^46^. Finally, the binding energy assessed the macromolecules' binding mode and classified them according to their coupling energies.

The docking model with the lowest energy score and the highest ligand root mean square deviation (RMSD) was selected for analysis of binding energy (Kd) scores using the PRODIGY server^47^. PRODIGY is a robust predictive system that uses the structural properties of protein–protein interactions, the number of interfacial contacts, and non-interacting surfaces to calculate the binding affinity of proteins^48^. In addition, the residual interactions of the three-dimensional model of the protein complexes were analyzed via the PDBsum server (http://www.ebi.ac.uk/pdbsum). Bound and unbound interacting residues between protein–protein interactions were examined.

### Molecular dynamics simulation

MD simulations were performed through the GROMACS 5.1.4 package^49^ using OPLS-aa force fields and the SP3 water model^50^. A dodecahedral box with 2 Å of freedom on the sides was utilized. The system was electrically neutralized with sodium ions. The Particle Mesh Ewald (PME) algorithm calculated the long-range electrostatic interaction. Subsequently, the energy minimization of all systems was computed using the steepest descent algorithm. The systems were then equilibrated using NVT and NPT with 400 ps steps, respectively, using a V-rescaled Berendsen thermostat and a Parrinello-Rahman NPT array progressively directed the equilibration of each system and the Nose–Hoover algorithm maintained temperature^51,52^. During NPT equilibrium, the Parrinello-Rahman barostat maintained pressures at 1 atm while the systems' heating gradually increased from 0 to 310 K. The MD simulation was conducted for the complexes in 25 ns. As a part of the analysis, we calculated root mean square deviation (RMSD), root mean square fluctuation (RMSF) of the Cα backbone, the secondary structure and the H-bonds evolution of the proteins using Gromacs tools. To estimate the electrostatic contacts, we calculate the number of interactions in the initial and final structure, using PDBsum.

### Supplementary Information

Supplementary Information 1.

Supplementary Information 2.

Supplementary Information 3.

Supplementary Tables.

## Data Availability

The datasets used and/or analyzed during the current study available from the corresponding author upon request. Please, see Supplementary Tables S1, S2 and S3 for a complete list of potential interacting partners identified by Y2H and details about this assay.
