# Supplementary material for: Experimental and computational evidence that Calpain-10 binds to the carboxy terminus of NaV1.2 and NaV1.6
Source: Sci Rep. 2024 Mar 21;14:6761. doi: 10.1038/s41598-024-57117-8 (PMC10957924; doi:10.1038/s41598-024-57117-8)
Supplement: Supplementary file 1 — Supplementary Information 1. [file 41598_2024_57117_MOESM1_ESM.pdf]

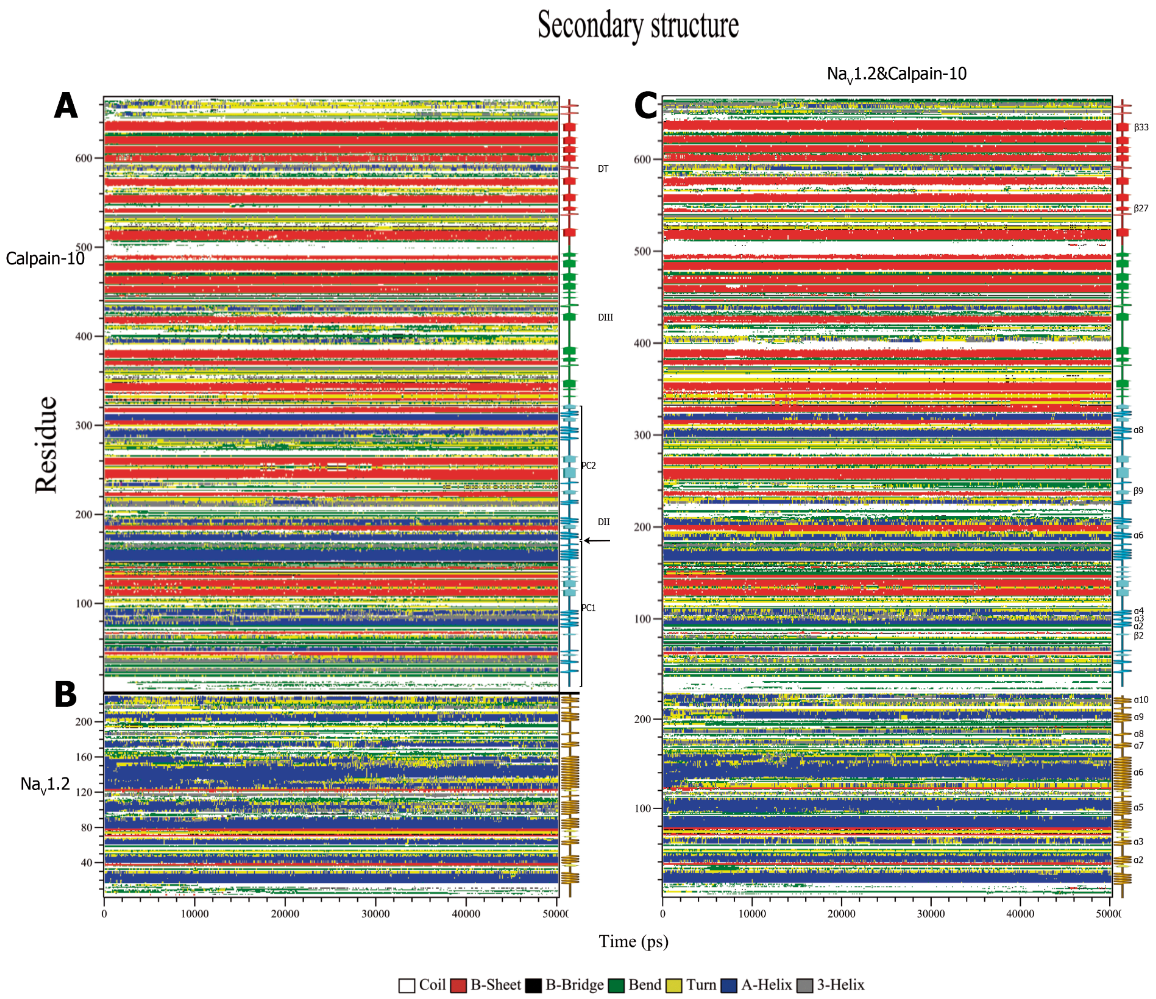

Supplementary Figure 1. Secondary structure evolution of Na_V1.2CT/Calpain-10. Right panel. The individual evolution of the secondary structure for Na_V1.2CT and Calpain 10. Calpain domains and Na_V1.2 initial secondary structure are shown on the left edge. **Left panel**. The evolution of the complex Na_V1.2CT/Calpain-10 is shown. On the left edge the alpha-helices and ß-sheets that become partially unfolded are labeled. PC1 alpha-helices 2, 3 4 and 6 belong to PC1 and PC2 motifs in proteolytic DII. Also DT ß-sheet7 and 33 show changes.

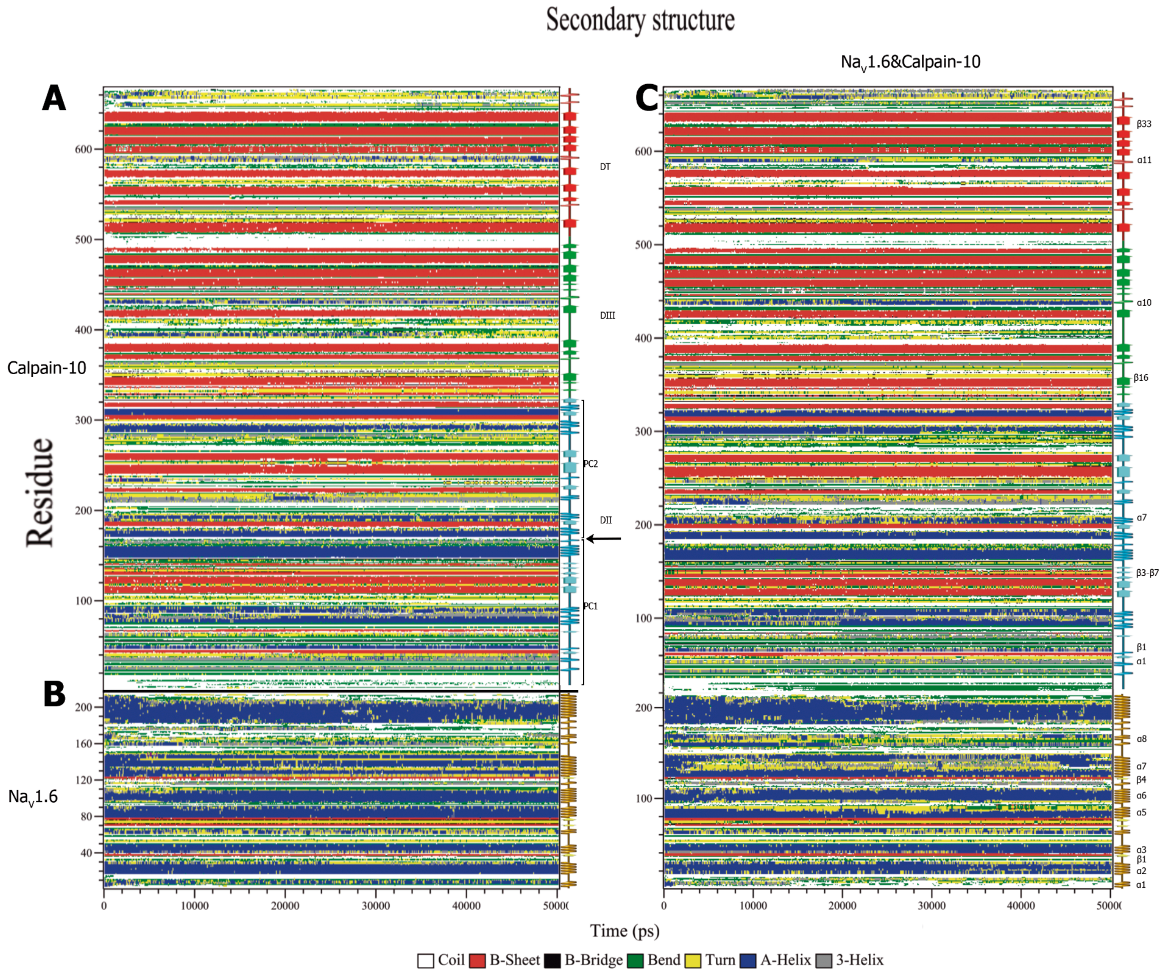

Supplementary Figure 2. Secondary structure evolution Na$_V$1.6CT/Calpain-10 complex. Right panel. The individual evolution of the secondary structure for Na$_V$1.6CT and Calpain 10. Calpain domains and Na$_V$1.6 initial secondary structure are shown on the left edge. **Left panel**. The evolution of the complex Na$_V$61.2CT/Calpain-10 is shown. On the left edge the alpha-helices and ß-sheets that become partially unfolded are labeled. Main changes are noticeable in DII alpha-helix10 and ß-sheet16, as well as ß-sheets3-7 within the PC1 motif in domain II. Also DT alpha-helix11 and ß-sheet33 show changes.
