# Supplementary material for: Experimental and computational evidence that Calpain-10 binds to the carboxy terminus of NaV1.2 and NaV1.6
Source: Sci Rep. 2024 Mar 21;14:6761. doi: 10.1038/s41598-024-57117-8 (PMC10957924; doi:10.1038/s41598-024-57117-8)
Supplement: Supplementary file 2 — Supplementary Information 2. [file 41598_2024_57117_MOESM2_ESM.pdf]

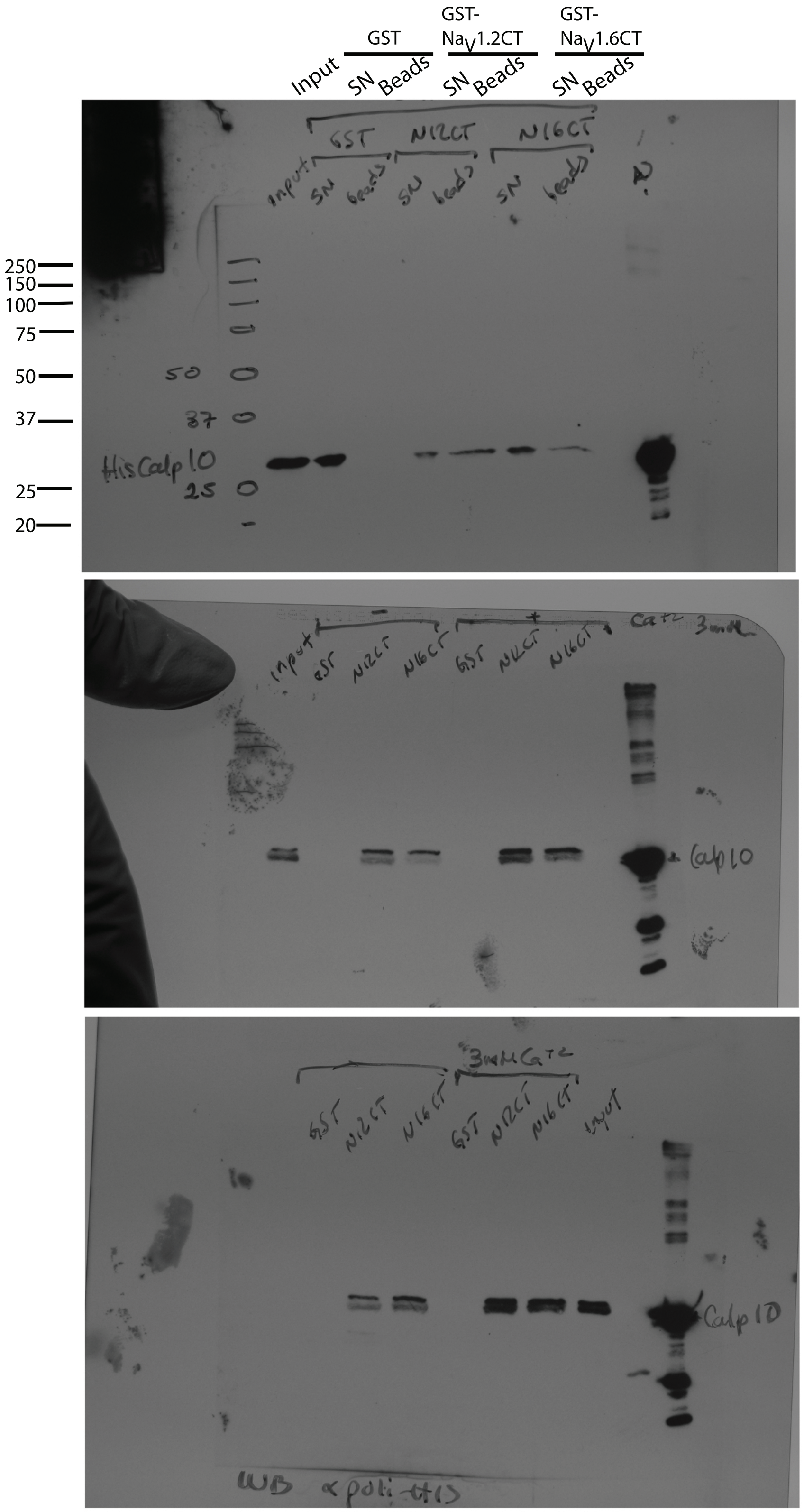

Supplementary figure 3. In vitro binding of His6Calpain-10 to the C-terminus of NaV1.2 and NaV1.6. Recombinant proteins GST, GST-NaV1.2CT or NaV1.6CT, bound to glutathione-beads, were incubated with recombinant His6-Calp10. After extensive washing, the proteins bound to glutathione-beads were analyzed by western blot with an anti-poly-Histidinde antibody. Proteins were separted by standard SDS-PAGE in a 12% Acrylamide gel with a pre-stained protein ladder. Anti-mouse HRP-conjugated antibodies were used to detect immunoreactivity of poly-his antibody. The HRP signal was revealed by chemiluminesence and captured on film (Amerhasm hyperlfim ECL) Exposure was minimized to prevent overexposure (1 to 3 minutes), therefore the edges of the membranes are only partially visible. The film was developed and anotated by hand, and then digitazed with a Canon PowerShot G5 camera. **Upper pannel** shows binding in absence of calcium (binding buffer: 50 mM Tris-HCl, pH 7.5, 120 mM NaCl, 2 mM EGTA, 0.1% triton X-100, 2 mM DTT). This blot was used to produce Figure 1, here the full lenght of the membrane is shown. A single band of ~29 kDa, matching the expected molecular weight of the His6-Calpain10, was identified, indicating that it is able to bind to GST-NaV1.2CT and GST-NaV1.6CT but does not bind to GST alone. A sample of His6-Calpain 10 produced in BL21 was loaded as a control in the most left lane.
The **middle and bottom pannels** show two more independent experiments were His6-Calpain-10 is pull-down by GST-NaV1.2CT or NaV1.6CT in presence or absence of 3 mM Ca2+. Here, only the proteins bound to the GST-sepahrose beads were electrophoresed.
