# Supplementary material for: Experimental and computational evidence that Calpain-10 binds to the carboxy terminus of NaV1.2 and NaV1.6
Source: Sci Rep. 2024 Mar 21;14:6761. doi: 10.1038/s41598-024-57117-8 (PMC10957924; doi:10.1038/s41598-024-57117-8)
Supplement: Supplementary file 3 — Supplementary Information 3. [file 41598_2024_57117_MOESM3_ESM.pdf]

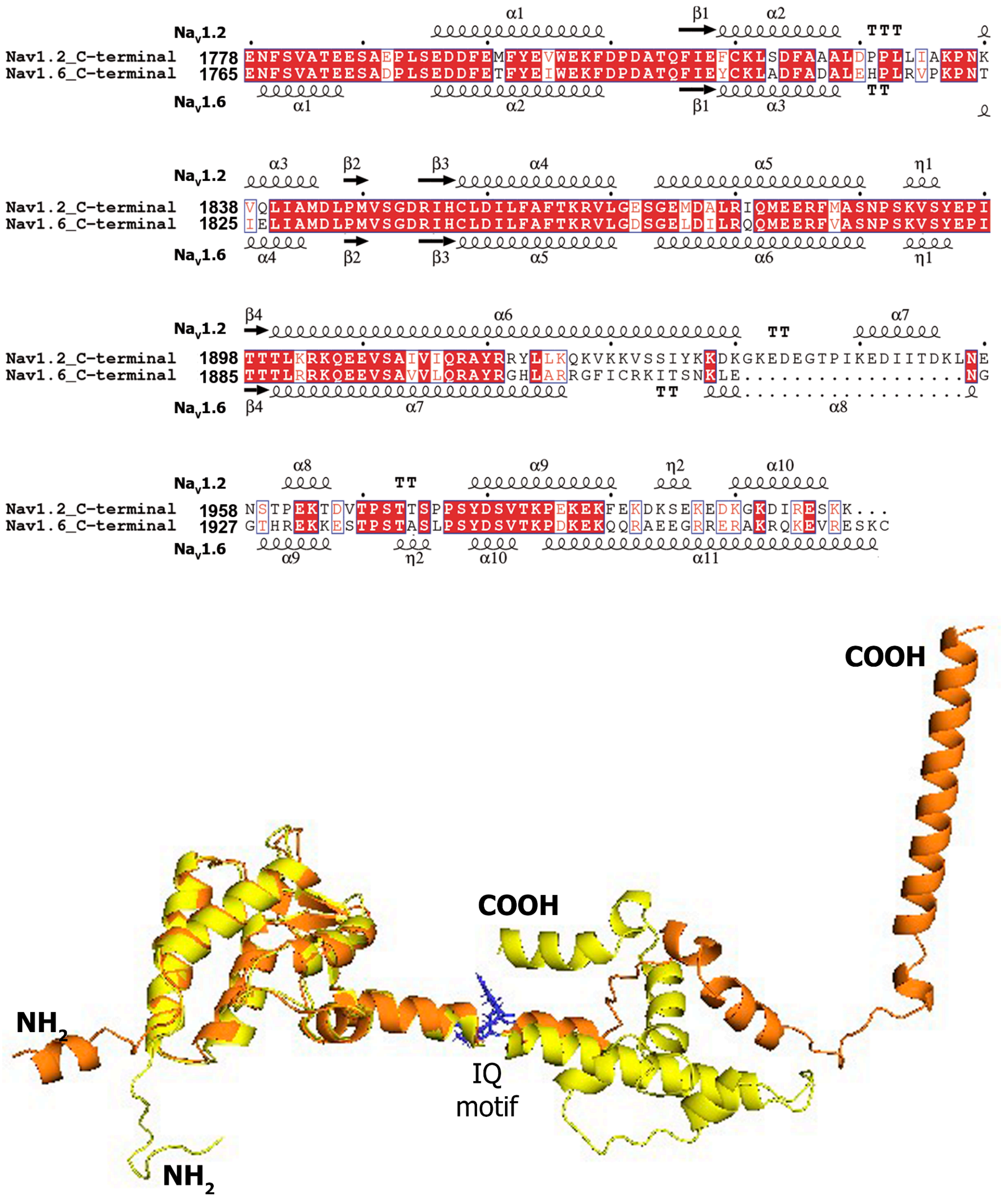

Supplementary Figure 4. Alignment between NaV1.2 and NaV1.6. Top panel: Alignment between NaV1.2 and NaV1.6 and secondary structure composition, identical amino acids are highlighted in red. Helixes are drawn as squiggles, β-strands as arrows, and turns with TT letters). Bottom panel: NaV1.2 (yellow) and NaV1.6(orange) overlapping, the IQ motif is shown in blue. RMSD= 1.306 A. RMSD was calculated with Pymol.

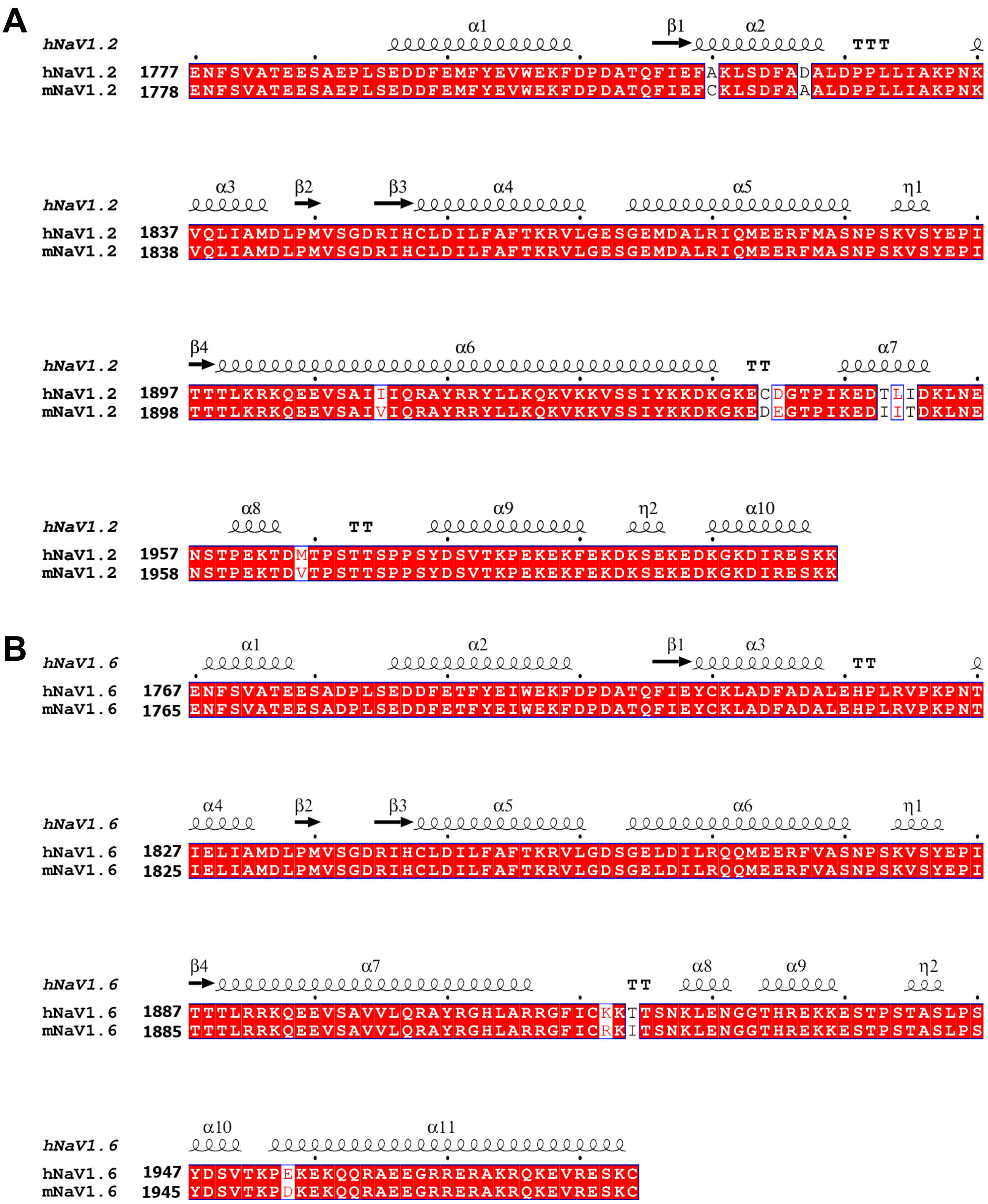

Supplementary Figure 5. C-terminus of mouse and human NaV1.2 and NaV1.6. Alignment between human and mouse NaV1.2(**A**) and NaV1.6 (**B**) are shown. Secondary structures are depicted above alignments. Helixes are drawn as squiggles, β-strands as arrows, and turns are labeled TT. Identical amino acids are highlighted in red.
