# Supplementary material for: Experimental and computational evidence that Calpain-10 binds to the carboxy terminus of NaV1.2 and NaV1.6
Source: Sci Rep. 2024 Mar 21;14:6761. doi: 10.1038/s41598-024-57117-8 (PMC10957924; doi:10.1038/s41598-024-57117-8)
Supplement: Supplementary file 4 — Supplementary Tables. [file 41598_2024_57117_MOESM4_ESM.pdf]

Supplementary table 1. Y2H summary

Bait	Cotransformation	Transformation efficiency (cfu/μg plasmid)	total cfu screened	total tested for His, Leu and Trp prototrophy	His+	LacZ+	
						time	#
C-terminus Nav1.2	3.01.05	1.26×10^4	1.26×10^6	~850	801	30 min-3h	271
	100 μg library/200μg bait					20 h	360
	28 plates/200 μl						
C-terminus Nav1.6	3.28.05	1.35×10^4	1.35×10^6	~760	505	30 min-3h	487
	100 μg library/200μg bait						
	33 plates/200μl						

The Initial screening was performed by cotransfecting L40 cells with the library and each of the different bait plasmids. The L40 cells are an haploid strain genetically modified to be unable to synthetize several aminoacids, including histidine, triptophan and leucine. The bait and library plasmids contain a functional copy of TRP1 and LEU2 genes, respectively, so that autotrophy for trp and leu is restored when cells are transfected with both plasmids. When the bait and library fusion proteins interact, the DNA-BD an the AD are brought into proximity, thus activating transcription of two reporter genes, HIS3 and LacZ. Clones developing a blue color in presence of X-Gal. Color developing was monitored 30 min, 3h and 20 h after exposition to X-Gal. Individual clones were replated in -Leu-His-Trp defined media and tested again for ß-galactosidaes activity, two or three times.

Curing. All the clones were further cured, this is plated on -Leu defined media to release the selection pressure for the TrpHis prototrophy confered by bait-plasmids. On time, those cells that had lost the bait plasmid turned pink due a mutation of the L40 ADE2 gene that results in the accumulation of a red pigment when cells are grown in a media with limited adenine. In addition to the TRP1 marker, the bait plasmids contains a non-mutated copy of ADE2 which allows the cells overcome the mutation, so they don't acumulate red pigment and remain white. Once cured, the cells were tested again for ß-galactosidase activity to eliminate clones with intrinsic transcriptional activity. Isolated clones were replated and tested again 2 or 3 times. Most of the clones that developed a blue color after 20h turn out to be calmoduline clones (many out of frame)

Supplementary Table 2. Putative Sodium Channels Interacting Proteins. Y2H output

Clone	NCBI Reference sequence	Putative interacting protein	Other names	ß-Galactosidase activity stronger with: Nav1.2 vs Nav1.6	Function
T19-1, T22-293	NM_134079.4 NM_001243041.1	Adenosine Kinase		Nav1.2	kinase
T19-8, T19-11	NR_157369.1 NM_145488.2	Peroxisomal biogenesis factor 6	PEX6	Nav1.2	AAA ATPase required for peroxisome fusion and/or peroxisomal matrix protein import.
T19-23	NM_057171.3	BCL2-associated athanogene 6 (Bag6)	Bat3, HLA-B associated transcript, Sycthe, HAP46/ BAG-1M	Nav1.2	Function is not well stablished yet, but there is some evidence for a role as apoptotic regulator. Sequence matches another 50 sequences on refseq_rna database, confrimed or prdicted, for Bag6 variants. seems to interact with laminin but not with CoRest or pincher.
T19-24	NM_021273.4	Creatine kinase, brain (Ckb)	Brain-type Creatin Kinase (B-CK)	Nav1.2	Regeneration of ATP from mitochondrial phosphocreatine.
T19-38	NM_007924.3	Mus musculus elongation factor RNA polymerase II (Ell), mRNA		Both	Elongation Factor
T19-41	NM_178405.3	Na+-K+ ATPase			
T19-43 and another 800 clones	NM_007589.5 NM_001355703.1	Calmoduline	CaM	Both	Calcium binding protein.

Clone	NCBI Reference sequence	Putative interacting protein	Other names	ß-Galactosidase activity stronger with: Nav1.2 vs Nav1.6	Function
T19-49	XM_006533811.4<br>XM_011249157.4<br>JN956676.1<br>JN948795.1<br>BC063781.1<br>BC058119.1<br>AK087537.1<br>AK083155.1<br>AK077568.1<br>AK051448.1<br>AK082083.1<br>AY158926.1<br>AL662809.14<br>AK015962.1<br>NM_178218.4<br>AK187267.1<br>AL589651.8<br>AY158911.1<br>NM_178183.2<br>BC117110.1<br>M33988.1	IgM	heavy chain of IgM	Nav1.2	immunoglobuline
T19-51	NM_011655.5	Tubulin ß5		Nav1.2	Main component of microtubules
T19-56	NR_185127.1<br>NM_001420051.1<br>NM_001420060.1<br>NM_001420052.1<br>NM_001420058.1<br>NM_001420053.1<br>NM_001420054.1<br>NM_001420048.1<br>NM_024168.3<br>NM_001420062.1<br>NM_001164205.2<br>NM_001164204.2<br>NM_001420061.1	Leucocyte receptor cluster member 5		Nav1.2	t-RNA splicing endonuclease. seems to interact with lamin A but not with CoRest or pincher

Clone	NCBI Reference sequence	Putative interacting protein	Other names	ß-Galactosidase activity stronger with: Nav1.2 vs Nav1.6	Function
T22-31	NM_001424196.1<br>NM_001368777.2<br>NM_001361936.2<br>NM_001284392.2<br>NM_001368775.2<br>NM_001368776.2<br>NM_001368779.2<br>NM_011129.3<br>NM_001368778.2<br>NM_001284394.2<br>NM_001284398.2	Sept4 protein	Septin, sep4, H5, hCDCerl -2a, hCDCrel 2b, Bradeion -alpha and – beta, ARTS, MART, Pnutl2	Nav1.2	GTPase. Septins participe in filament formation, citokinesis and seem to be involved in vesicular trafficking.
T22-47, T22-121, T22-269, T22-314, T22-395, T22-446	NM_001358359.1	Nuclear Receptor Binding Protein 2 (nrbp2)	2-2.6 kb	Both	Function is unknow, but the closely related protein nrbp its tougth to be involved in protein trafficking between ER and Golgi
T22-53, T22-561	NM_008319.3<br>NM_001410238.1	Intracelullar adhesion molecule 5	ICAM5, Telencep halin (TLN)	Both	Promote dendritic outgrowth.
T22-54	NM_175519.5	Hypothetical protein	1.5 kb	Nav1.2	Unknow. Similar to K+ channel tetramerization domain
T22-65	NM_010201.5<br>NM_207667.4	Fibroblast growth factor homologous 4 isoform A (FHF4A)	Fibrobla st growth factor 14 (FGF14) variant1	Both	Unclear, but this and other members of this FGF subfamily have been reported to interact with Na Channels and MAP kinase proteins.

Clone	NCBI Reference sequence	Putative interacting protein	Other names	ß-Galactosidase activity stronger with: Nav1.2 vs Nav1.6	Function
T22-88	NM_013415.6	Na^+-K^+ ATPase ß2 subunit		Nav1.2	Ionic Pump auxiliary subunit
T22-131, T22-702, T22-713, T22-748	NM_009188.4 NM_001113248.2	Sin3B	UME4, RPD1, GAM2, CPE1, SDS16	Both	Provide structural support for an heterogenouos Sin3/histone deacetylase complex
T22-137, T22-339, T22-539	NM_009721.6	Na^+-K^+ ATPase ß1 subunit		Both	Auxiliay subunit. Necesary to properly folding of the N-K ATPase alfa subunit
T22-198	NM_175244.4	Hypothetical protein. HECT domain protein, C-terminus 41aa		Na1.2	Predicted ubiquitin-protein ligase activity
T22-205	NM_028211.1	Hypothetical protein		Both	Unknow
T22-291	NM_028469.4	Hypothetical protein		Both	Unknow
T22-322	NM_011796.2	Calpain 10		Nav1.2	calcium-activated neutral proteinase
T22-342	NM_008064.4 NM_001159324.2	Lysosomal α-glucosidase		Nav1.2	Hydrolysis of terminal, non-reducing 1,4-linked alpha-D-glucose residues with release of alpha-D-glucose
T22-368	NM_010918.2	Hypothetical protein		Nav1.2	Unknow
T22-485	NM_009087.2 NM_181730.4	RNA polymerase 1-3		Nav1.2	RNA polymerase

Clone	NCBI Reference sequence	Putative interacting protein	Other names	ß-Galactosidase activity stronger with: Nav1.2 vs Nav1.6	Function
T22-533	NM_001029979.4<br>NM_001414452.1<br>NM_001355463.2<br>NM_001414451.1<br>NM_001414453.1<br>NM_001414450.1<br>NM_001414449.1<br>NM_001414454.1	Scaffold attachment factor B2	SAFB2, HET, HAP	Both	Unclear. Cytoplasmic protein, may be involved in tumurogenesis
T22-611	NM_001357688.1<br>NM_001163488.1<br>NM_001163487.1<br>NM_021514.4	Phosphofructokinase	Pfkm	Nav1.2	kinase
T22-657a	NM_009679.3<br>NM_001302970.1	Adaptor protein μ1	Ap2m1	Nav1.2	adaptor-related protein complex 2, mu 1 subunit isoform b
T22-717	NM_001080706.1	BTAFI	Tata binding associated protein factor BTAF1 RNA polymerase II, B-TFIID transcription factor-associated	Nav1.2	Transcription factor
T22-730	NM_053183.3<br>NM_001358819.1<br>NM_001358820.1	RNA Helicase	DEAD protein	Both	Helicase

Clone	NCBI Reference sequence	Putative interacting protein	Other names	ß-Galactosidase activity stronger with: Nav1.2 vs Nav1.6	Function
T22-737, T22-442	NM_001131020.1<br>NM_010277.3	GFAP	Glial fibrillary acidic protein	Nav1.2	Main component of intermediate microfilaments in astrocytes
T22-785	NM_001421575.1<br>NM_001310535.1<br>NM_001198861.1<br>NM_001198860.1<br>NM_001198859.1<br>NM_013502.3	HIRIP 5	C-terminus binding protein 1	Nav1.2	Trasncription factor. Also have dehidrogenase activity. Some splicing variants have a role in other biological processes. It shuttles between nucleous and cytoplasma
T22-816	NM_001170591.1<br>NM_020045.3	CTBP1	Histone cell cycle regulation defective interacting protein. Nfu	Nav1.2	Posses a NifU-like domain, involved in the biosynthesis of Fe–S clusters. Scaffold protein

This table contains a depurate list of putative interacting proteins identified through the yeast two hybrid assay (Y2H). After the ininial selection, cells were cured as described above. Then, to eliminate false postives we mated the pre-selected clones with another yeast strain (AMR70) pre-transformed with each of several plasmids encoding unrelated proteins in fusion with DNA-BD. AMR70 cells pre-transformed with either of the original baits (Nav1,2CT, Nav1.6CT, Nav1.2L2 or Nav1.6L2) or plasmids encoding unrelated proteins (Laminin, CoREST and pincher). The AMR70 cells genotype is very similar to the L40, except that does not contains the HIS3 reporter gene. So, the resultant diploides were selected by using the Leu and Trp markers, and we relied in the ß-galactosidase activity to test for interaction between the pre-selected and the bait fusion proteins. We eliminated all those clones that turned blue in 3h or less in presence of laminin, CoREST or Pincher. We also eliminate those that were unable to reproduce the interaction with any of both sodium channels baits. Thus we reduced the number of clones from 1,118 to 34 putative interacting partners. T19 and T22 clones were screened with the C-terminus of mouse Nav1.2 (pSN12CT). T20 and T23 clones were screened with the C-terminus of mouse Nav1.6 (pSN16CT). All clones listed here were His+ and LacZ+ in 30-3h during the initial screening.
